# SNARE VTI13 plays a unique role in endosomal trafficking pathways associated with the vacuole and is essential for cell wall organization and root hair growth in arabidopsis

**DOI:** 10.1093/aob/mcu041

**Published:** 2014-04-15

**Authors:** Emily R. Larson, David S. Domozych, Mary L. Tierney

**Affiliations:** 1 Cellular, Molecular and Biomedical Science Program; 2 Department of Plant Biology, University of Vermont, Burlington, VT, USA; 3 Department of Biology, Skidmore College, Saratoga Springs, NY, USA; 4 Institute of Molecular Cell and Systems Biology, University of Glasgow College of Medical, Veterinary & Life Sciences, Glasgow G12 8QQ, UK

**Keywords:** *Arabidopsis thaliana*, root hairs, tip growth, vesicle trafficking, SNARE proteins, early endosomes, plant cell wall, vacuole, membrane transport, endosomal trafficking pathway, trans-Golgi

## Abstract

**Background and Aims:**

Root hairs are responsible for water and nutrient uptake from the soil and their growth is responsive to biotic and abiotic changes in their environment. Root hair expansion is a polarized process requiring secretory and endosomal pathways that deliver and recycle plasma membrane and cell wall material to the growing root hair tip. In this paper, the role of VTI13 (AT3G29100), a member of the VTI vesicular soluble NSF attachment receptor (SNARE) gene family in *Arabidopsis thaliana*, in root hair growth is described.

**Methods:**

Genetic analysis and complementation of the *vti13* root hair phenotypes of *Arabidopsis thaliana* were first used to assess the role of VTI13 in root hair growth. Transgenic lines expressing a green fluorescent protein (GFP)–VTI13 construct were used to characterize the intracellular localization of VTI13 in root hairs using confocal microscopy and immunotransmission electron microscopy.

**Key Results:**

VTI13 was characterized and genetic analysis used to show that its function is required for root hair growth. Expression of a GFP–VTI13 fusion in the *vti13* mutant background was shown to complement the *vti13* root hair phenotype. GFP–VTI13 localized to both the vacuole membrane and a mobile endosomal compartment. The function of VTI13 was also required for the localization of SYP41 to the *trans*-Golgi network. Immunohistochemical analysis indicated that cell wall organization is altered in *vti13* root hairs and root epidermal cells.

**Conclusions:**

These results show that VTI13 plays a unique role in endosomal trafficking pathways associated with the vacuole within root hairs and is essential for the maintenance of cell wall organization and root hair growth in arabidopsis.

## INTRODUCTION

Root hairs perform an important role in vascular plants in the response to environmental stimuli (Shin *et al*., 2005). Their growth and shape are largely dependent on the secretion and assembly of new plasma membrane and cell wall materials at the tip of the cell (Campàs and Mahadevan, 2009; Cárdenas, 2009). The root hair is a specialized extension of the root epidermis, where all new plasma membrane and cell wall components are secreted to the expanding tip of the cell. This polarized growth mechanism is dependent on targeted secretion of vesicles to the root hair apex as well as endosomal pathways that recycle membrane and cell wall material from the plasma membrane and apoplast (Grebe *et al*., 2003; Ketelaar *et al*., 2008; Barberon *et al*., 2011). However, while vesicle transport pathways to and from the root hair tip are known to be required for growth, the players in these pathways are still poorly resolved.

Vesicular **S**oluble **N**SF **A**ttachment **Re**ceptor (SNARE) proteins are necessary for the fusion of cargo-laden vesicles to target compartment membranes involved in endosomal pathways (Grefen and Blatt, 2008). In plants, SNAREs are involved in the transport of plasma membrane and cell wall components to sites of active cell expansion, as well as in the intracellular transport of proteins required for plasma membrane and organelle function (Heese *et al*., 2001; Silva *et al*., 2010; De Caroli *et al*., 2011). SNARE proteins have been identified as important players for secretion and embryonic development (Lukowitz *et al*., 1996; Assaad *et al*., 2004). Co-localization studies in plants have also linked subsets of SNAREs with other types of transport proteins associated with secretory and endosomal pathways (Uemura *et al*., 2004; Guo and McCubbin, 2012; Silva *et al*., 2010), providing further evidence that SNARE function in vesicle trafficking is important for plant growth.

The VTI-type SNAREs were first identified in plants based on homology to the essential yeast VTI1a SNARE (Fischer von Mollard and Stevens, 1999; Zheng *et al*., 1999) and include four family members: VTI11, VTI12, VTI13 and VTI14 (Surpin *et al*., 2003). While the VTI-type SNAREs in plants are likely to have similar functions in endosomal trafficking as in yeast, the increased number of VTI-related SNAREs in plants suggests the possibility of neo-functionalization of one or more family members (Sanmartín *et al*., 2007). Genetic analysis of VTI SNARE function in arabidopsis has shown that *vti11*/*zig1* null mutants exhibit a zig-zag growth pattern of the inflorescence stem, a shoot agravitropic response and defects in *trans-*Golgi network (TGN) and vacuole trafficking pathways (Kato *et al*., 2002; Niihama *et al*., 2005, 2009). In comparison, *vti12* mutants lack these growth phenotypes but are more sensitive to nutrient deprivation and senesce faster than wild-type or *vti11* mutants (Surpin *et al*., 2003). Early-onset senescence has been linked to arabidopsis autophagy mutants (Doelling *et al*., 2002; Hanaoka *et al*., 2002), and expression of early senescence in *vti12* but not *vti11* further supports a divergence of the functions of these two family members in plants.

The unique functions of VTI11 and VTI12 are attributed to their formation of SNARE complexes with different syntaxin proteins and their localization to distinct intracellular membranes (Bassham *et al*., 2000; Sanderfoot *et al*., 2001*a*; Surpin *et al*., 2003). VTI11 has been shown to accumulate within the vacuole and prevacuole membranes, while VTI12 localizes to the TGN and plasma membrane (Surpin *et al*., 2003; Uemura *et al*., 2004). In addition, VTI11 and VTI12 have been shown to be required for transport of distinct cargos to the lytic and storage vacuoles, respectively (Sanmartín *et al*., 2007). These data support the model that VTI11 and VTI12, while closely related at a sequence level, have different functions in endosomal transport processes. The importance of these pathways is underlined by the embryo-lethal phenotype of the *vti11 vti12* double mutant, indicating that these SNAREs contribute to endosomal processes required for plant viability (Surpin *et al*., 2003).

By comparison, there is little information describing the functions of VTI13 or VTI14 in plants. A single expressed sequence tag clone has been identified for VTI13, suggesting that it is expressed in plants, and work in arabidopsis cell culture has shown that a green fluorescent protein (GFP)–VTI13 fusion protein localizes to membranes of the vacuole and prevacuolar compartment (Uemura *et al*., 2004). In this paper we provide genetic evidence that VTI13 is required for normal root hair growth and that expression of a GFP–VTI13 fusion protein in the *vti13* background is sufficient to complement the mutant root hair phenotypes. Confocal analysis of the GFP–VTI13 fusion protein in transgenic plants provides evidence for a role for VTI13 both in trafficking of cargo to the vacuole and in TGN/early endosome organization and function in root hairs. Lastly, analysis of cell wall organization and root hair growth in *vti13* and the *prp3 vti13* double mutant supports a model for VTI13 in the assembly or maintenance of the root hair cell wall.

## MATERIALS AND METHODS

### Plant materials and growth conditions

*Arabidopsis thaliana* (Columbia-0) was used for all experiments involving wild-type and mutant analysis. Our standard plant growth media for seedlings consisted of 1× Murashige and Skoog (MS) salts, 1 % (w/v) sucrose, 1 × Gamborg's vitamin solution, 5 mm 4-morpholineethanesulfonic acid sodium salt, pH 6, and 1·3 % (w/v) agarose (Sigma Chemical, St Louis, MO, USA). Sterilized seeds were grown vertically on plates for 5 days at room temperature under continuous light. Other growth conditions included the addition of 200 mm mannitol to the standard medium and changing the pH of the medium from 6·0 to 5·0. For plants grown in soil, seed was sown in soil (MetroMix 360, Sun Gro Horticulture, Bellevue, WA, USA) and placed in growth chambers (Conviron, Winnipeg, CA, USA) programmed for long-day conditions (16 : 8 h light:dark cycle, 20 °C).

### RNA isolation and RT-PCR

Seedlings were grown on our standard medium for 5 days, after which root tissue was harvested for RNA isolation. Approximately 200 roots were pooled per genotype for each condition tested and duplicate analyses were performed. When harvesting the root tissue, the root meristem and mature region of the root were removed such that only the differentiation and elongation zones of the root were collected. Total RNA was isolated using the Qiagen RNeasy Plant Mini Kit protocol and then used in first-strand cDNA synthesis using SuperScript II Reverse Transcriptase according to the standard protocol (Invitrogen). For RT-PCR the VTI13 forward and reverse primers described in Supplementary Data Table S1 were used with the first-strand cDNA templates to amplify gene products using Phusion Taq polymerase (New England Biolabs).

### Generation of GFP–VTI13 constructs

#### 35S:GFP–VTI13 construct

A 35S:GFP–VTI13 construct was kindly provided by Dr Masa Sato (Uemura *et al*., 2004) and was subcloned into the pENTR/D-TOPO vector (Invitrogen, Grand Island, NY) to use in Gateway cloning. pENTR/35S:GFP–VTI13 (pERL02A) was cloned into the Gateway destination vector pEarleyGate CDS694 (Earley *et al*., 2006), using the LR Clonase II reaction kit (Invitrogen) according to the manufacturer's directions. Clones containing 35S:GFP–VTI13 in the CDS694 vector were transformed into *Agrobacterium tumefaciens* strain GV3101, after which the 35S:GFP–VTI13 construct (pERL02) was transformed into arabidopsis using the floral dip method (Bechtold and Pelletier, 1998; Zhang *et al*., 2006).

#### VTI13:GFP–VTI13 construct

A 2-kb DNA fragment just upstream of the *VTI13* translational start codon and including the 5′-UTR was amplified from genomic DNA using primers that added BamHI and NcoI restriction enzyme sites at the 5′ ends (VTI13pro_F and VTI13pro_R). This PCR product was then subcloned into the pENTR/D-TOPO entry vector (Invitrogen) using the manufacturer's protocol to produce pERL03A. This construct and pERL02 were digested with both BamHI and NcoI and then gel purified to extract the 2-kb VTI13 promoter fragment from pERL03A and the promoterless pERL02 linear plasmid. The VTI13 promoter was inserted upstream of the GFP–VTI13 construct using standard molecular techniques. The *VTI13*:GFP–VTI13 construct (pERL03) was first transformed into *Escherichia coli* strain DH5α and then into *A. tumefaciens* strain GV3101 for the transformation of arabidopsis.

### Characterizing *vti13* root hair phenotypes

Seeds were sterilized and plated on 1× MS medium, pH 6, 1× MS medium, pH 5, or 1× MS medium, pH 6, with the addition of 200 mm mannitol, and grown as described above. To characterize root hair morphology, seedlings were mounted in sterile distilled water or 1× MS liquid medium on glass slides. Images of root hairs were obtained using a Nikon Eclipse TE200 inverted microscope in conjunction with SPOT imaging software (Diagnostic Instruments). Root hair lengths were measured from bright-field images using the calibrated measuring tool in the SPOT imaging software. Statistical analysis (ANOVA) was performed using Prism 6·0.

### Complementation of *vti13* mutant phenotypes

A single T-DNA insertion mutant (SALK_075261) of *VTI13* was obtained from the Arabidopsis Biological Research Center (ABRC) stock centre. Primers used for genotyping this mutant are listed in Supplementary Data Table S1 and spanned all of the introns within *VTI13*. The *vti13* mutant was transformed with both the 35S:GFP–VTI13 and *VTI13*:GFP–VTI13 constructs. Homozygous transgenic lines expressing each *VTI13* construct were identified through glufosinate (BASTA) resistance in the *T*_3_ generation and then analysed for complementation of the *vti13* short, branched root hair phenotype in 5-day-old seedlings germinated on 1× MS solid medium. Root hairs were imaged using bright-field microscopy.

### Producing the *prp3 vti13* double mutant

The *vti13* homozygous null mutant was crossed to a *prp3* homozygous null mutant and *F*_1_ seed was collected. Individual *F*_2_ plants were genotyped using primer sets listed in Supplementary Data Table S1 to identify homozygous *prp3 vti13* double mutants. Several *prp3 vti13* double mutants from two independent crosses were characterized for their patterns of root hair growth in these studies.

### Latrunculin B and oryzalin treatments of arabidopsis seedlings

Arabidopsis seedlings were grown for 5 days on plates containing 1× MS medium, pH 6, and then transferred to the wells of a 48-well microtitre plate containing 200 μl of DMSO, 100 nm Latrunculin B or 10 μm oryzalin diluted in 1× MS medium, pH 6. Seedlings were incubated for 2 h at room temperature before confocal imaging of root hairs or root epidermal cells expressing 35S:GFP–VTI13. Seedlings were suspended in the treatment medium during imaging.

### Brefeldin A treatment of arabidopsis seedlings

Seedlings were grown for 5 days on 1× MS plates and then incubated with 10 or 50 μm brefeldin A (BFA) diluted in 1× MS medium directly on microscope slides. Seedlings treated with BFA were incubated at room temperature for 10 min before confocal imaging of GFP–VTI13. During imaging, seedlings were mounted in the treatment solutions. Where indicated, the lipophilic dye FM4-64 was added to the BFA treatment solutions at a concentration of 5 μm.

### Construction of transgenic lines expressing both yellow fluorescent protein–γ- tonoplast intrinsic protein and GFP–VTI13

To produce plants that co-expressed GFP–VTI13 and yellow fluorescent protein–γ-tonoplast intrinsic protein (YFP-γTIP), a transgenic line expressing 35S:GFP–VTI13 in a wild-type background was crossed with the 35S:YFP-γTIP transgenic line (Nelson *et al*., 2007), and heterozygous *F*_1_ seedlings were analysed by confocal microscopy.

### Confocal microscopy of GFP–VTI13 transgenic seedlings

All fluorescent lines were imaged with a Zeiss confocal LSM 510 META laser scanning microscope using a 63× oil immersion objective. The GFP–VTI13 fusion lines were excited with an argon ion laser line main beam splitter of 488 nm and emissions were captured using a 505/530 nm bypass filter. When FM4-64 staining was imaged with the GFP–VTI13 lines, the samples were excited at 488 nm with the argon ion laser with a main beam splitter of 488 nm and an additional splitter of 545 nm. The 505/530 nm bypass filter was used to capture GFP emission and a 650 nm long-pass filter was used to capture the FM4-64 emission. YFP–protein fusions were excited with a main beam splitter of 548/514 nm and emissions were captured with a 530/600 nm bypass filter. Cross-talk between YFP and GFP emission was taken into account by controlling the gain of each bypass filter orientation (as described in Atmodjo *et al*., 2011 and Supplementary Data Fig. S4). The LSM 510 META software was used to acquire images and movies of root hair and epidermal cells. Images were then processed using ImageJ.

### Seedling fixation for transmission electron microscopy

Roots were fixed in 1 % glutaraldehyde in Sorensen's buffer (EMS, Fort Washington, PA, USA) on ice for 45 min. The roots were subsequently washed three times with cold Sorensen's buffer and separated into two sets. One set was gently post-fixed in 0·8 % osmium tetroxide for 90 min on ice, washed three times with Sorensen's buffer, dehydrated in acetone and infiltrated/embedded in Spurr's Low Viscosity plastic. Polymerization of plastic was at 60 °C for 8 h. The second set of roots was not post-fixed but dehydrated in ethanol and infiltrated/embedded in London Resin. These procedures were performed at 4 °C and polymerization was performed with UV light at 4 °C for 16 h. Sections (60 nm) of roots were obtained using a Reichert Ultracut ultramicrotome and diamond knife. Sections were collected on Formvar-coated nickel slot grids. Immunogold labelling was performed as follows, using monoclonal antibodies against GFP (Invitrogen) or SYP41 (a generous gift from Natasha Raikhel). Grids containing osmicated specimens were treated for 5 min with 5 % H_2_O_2_ and then washed with distilled water. All grids were treated with 0·2 % ammonium chloride for 10 min, washed with distilled water and then incubated in a blocking solution consisting of 1 % bovine serum albumin in phosphate-buffered saline (pH 7·2) containing 0·1 % Tween-20 (PBST) for 30 min. Grids were washed and placed on droplets of a solution containing a 1/50 dilution of antibodies (SYP41 or GFP) in PBST for 90 min at 37 °C. After this time, the grids were washed and blocked again as described above. The grids were then incubated in a 1/150 dilution of anti-rabbit antibody in PBST for 90 min at 37 °C. The grids were washed with distilled water and then conventionally labelled with uranyl acetate and lead citrate. Sections were viewed with a Zeiss Libra 120 transmission electron microscope (TEM; Zeiss).

## RESULTS

### VTI13 is required for arabidopsis root hair growth

**Fig. 1.**
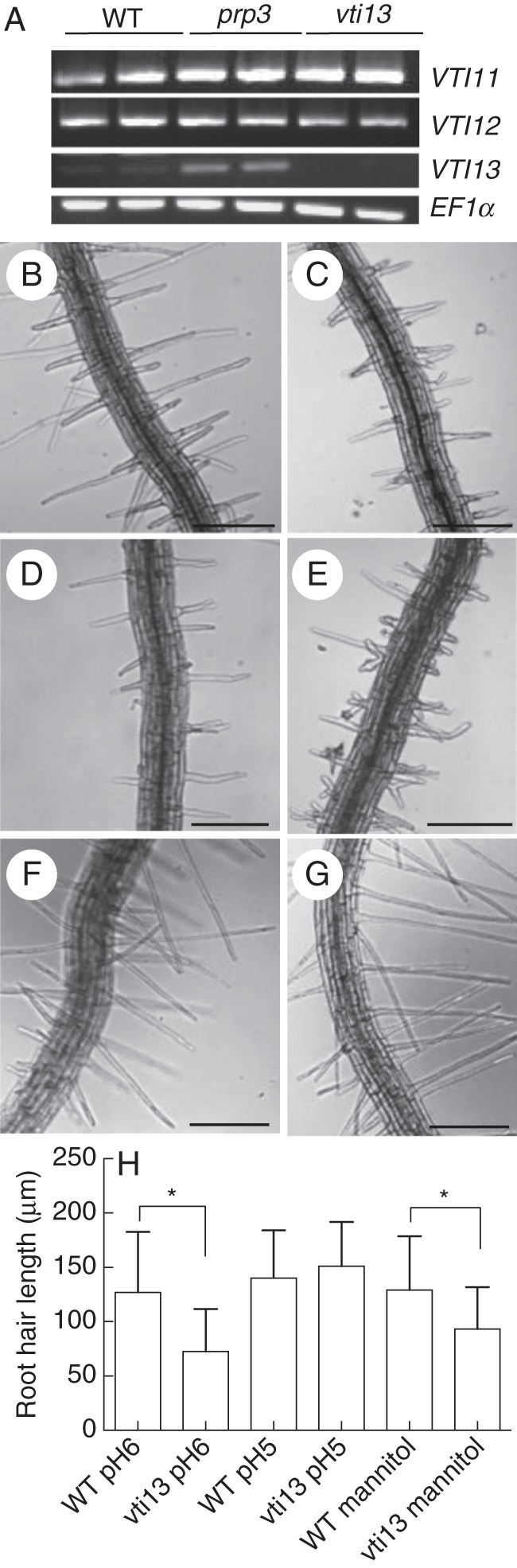
*VTI13* expression is elevated in *prp3* and is required for root hair growth. (A) Expression of the *VTI*-type SNARE family and *EF1-α* in wild-type (WT), *prp3* and *vti13* mutant seedling roots. (B–G) Comparison of root hair phenotypes of 5-day-old seedlings for (B) wild-type and (C) *vti13* on 1× MS, pH 6, (D) wild-type and (E) *vti13* on 1× MS, pH 6, plus 200 mm mannitol and (F) wild-type and (G) *vti13* on 1× MS, pH 5. (H) Average root hair lengths of seedlings grown in different growth medium conditions (*n* = 12 seedlings per condition). Asterisks denote statistical significances of differences in average root hair length compared with wild-type seedlings grown on the same media, using one-way ANOVA analysis (α = 0·01). Results are from one representative experiment and are given as the mean ± s.e. Scale bars: (B–G) = 50 μm.

The expression of the structural cell wall protein proline-rich protein 3 (PRP3; AT3G62680) is linked to root hair development (Bernhardt and Tierney, 2000), where it is localized to root hair cell walls and is required for root hair growth in arabidopsis (Hu *et al*., unpubl. res.). Microarray analysis of the T-DNA insertion mutant *prp3* identified *VTI13(*(*At3G29100*) as a gene whose expression is up-regulated in roots of *prp3* when compared with wild-type roots (Table 1). RT-PCR analysis was used to confirm the microarray results (Fig. 1A) and showed that *VTI13* transcripts were detected at very low levels in wild-type roots but were detected at higher levels in roots of the *prp3* mutant (Fig. 1A, Supplementary Data Fig. S1). These results suggest that VTI13 expression may be sensitive to root hair cell wall structure.

**Table 1.** Changes in gene expression of VTI-type SNARE family members observed in a microarray analysis in *prp3* roots normalized to gene expression values in wild-type roots

Accession number	Gene	Adjusted *P*-value	Log fold change
AT3G29100	*VTI13*	3·40E-05	2·20
AT5G39510	*VTI11*	0·7198	−0·128
AT1G26670	*VTI12*	0·13088	0·656
AT3 G62680	*PRP3*	5.10E-08	−7·00

*PRP3* expression is included as a control.

To determine if VTI13 was required for root hair growth, we analysed *vti13* (SALK_075261), which contains a T-DNA insertion in the second exon of the *VTI13* gene. Using RT-PCR, we could not detect any *VTI13* transcript in the *vti13* mutant, indicating that this line is a null mutant (Fig. 1A). In contrast, we found no discernable difference in the amount of transcript encoding *VTI11* or *VTI12*, two other VTI family members, in the *vti13* mutant (Fig. 1A, Table 1). To determine if the loss of VTI13 resulted in root hair growth phenotypes, *vti13* and wild-type seedlings were grown for 5 days on MS medium and examined using bright-field microscopy. In comparison with wild-type, the root hairs on *vti13* seedlings were shorter and exhibited branching (Fig. 1B, C). We then asked whether *vti13* root hair growth was sensitive to changes in the growth environment by comparing *vti13* root hair growth with that of wild-type seedlings in response to two other medium conditions. When grown in the presence of 200 mm mannitol, *vti13* seedling root hairs exhibited a much more severe root hair branching phenotype than that detected in wild-type or in *vti13* grown in the absence of mannitol (Fig. 1D, E). These results indicate that VTI13 is required for polarized growth in root hairs and that the *vti13* phenotype can be enhanced by osmotic stress. In contrast, when wild-type and *vti13* seedlings were grown on MS medium at pH 5·0, root hair length of wild-type seedlings was similar to that observed at pH 6, whereas the root hairs of *vti13* seedlings were longer and did not branch (Fig. 1F, G), suggesting that low pH may be able to compensate for factors altering *vti13* root hair growth.

**Fig. 2.**
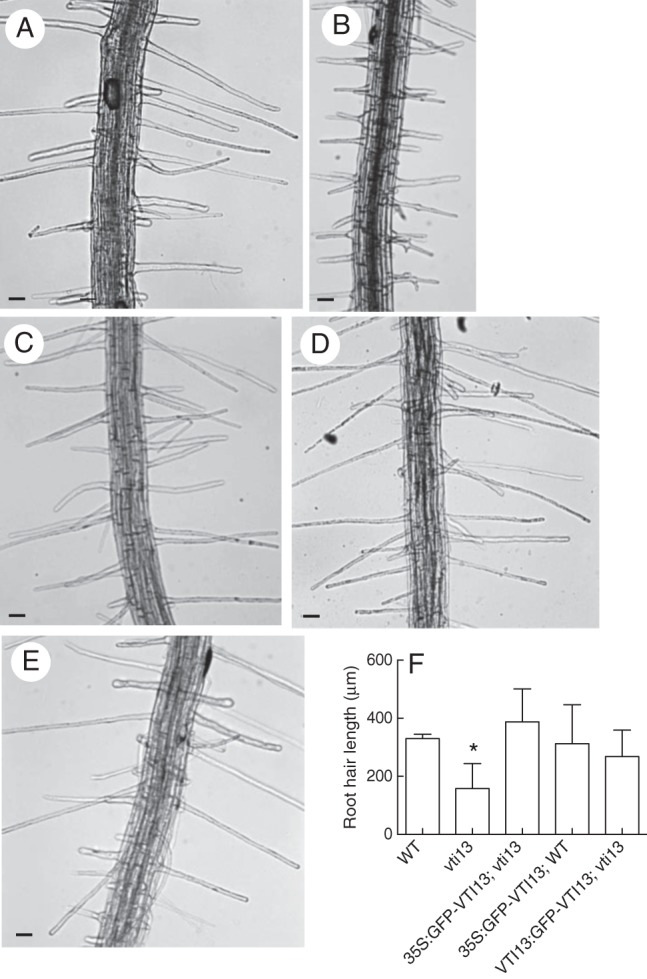
Mutant *vti13* can be complemented by the GFP–VTI13 fusion protein. Seedlings were grown for 5 days on 1× MS medium, pH 6. Root hairs of (A) wild-type, (B) *vti13* and (C) *T*_3_ seedlings of complemented *vti13* lines transformed with *VTI13*:GFP–VTI13 and (D) *T*_3_ seedlings of *vti13* transformed with 35S:GFP–VTI13. (E) The 35S:GFP–VTI13 construct expressed in the wild-type background. (F) Average root hair lengths of *vti13* and transgenic lines compared with wild-type root hairs using one-way ANOVA (α = 0·01). Results shown are from one representative experiment and represent the average of six to ten seedlings for each genotype and are given as the mean ± s.e. Scale bars: (A–E) = 50 μm.

To further confirm that the *vti13* root hair mutant phenotypes were due to the absence of VTI13, we examined root hair growth in *T*_3_ transgenic seedlings expressing either a 35S:GFP–VTI13 or a *VTI13*:GFP–VTI13 construct in the *vti13* mutant background (Fig. 2). Four to five *T*_3_ lines that were BASTA-resistant and showed similar expression in the *vti13* and wild-type backgrounds were identified for each construct. From these initial studies, one transgenic line expressing either 35S:GFP–VTI13 or *VTI13*:GFP–VTI13 was chosen for further analyses. Expression of the GFP–VTI13 protein fusion driven by either promoter complemented the *vti13* mutant root hair phenotype (Fig. 2C, D). Additionally, root hair growth was not affected by expression of the 35S:GFP–VTI13 construct in wild-type seedlings (Fig. 2E). Lastly, when the complemented *vti13* lines were grown in the presence of 200 mm mannitol, their root hair growth was indistinguishable from that of wild-type root hairs (Supplementary Data Fig. S2). These results indicate that the GFP–VTI13 constructs complemented the *vti13* root hair phenotypes and that VTI13 is essential for normal root hair growth.

### VTI13 localizes to the vacuole membrane in root hair cells

**Fig. 3.**
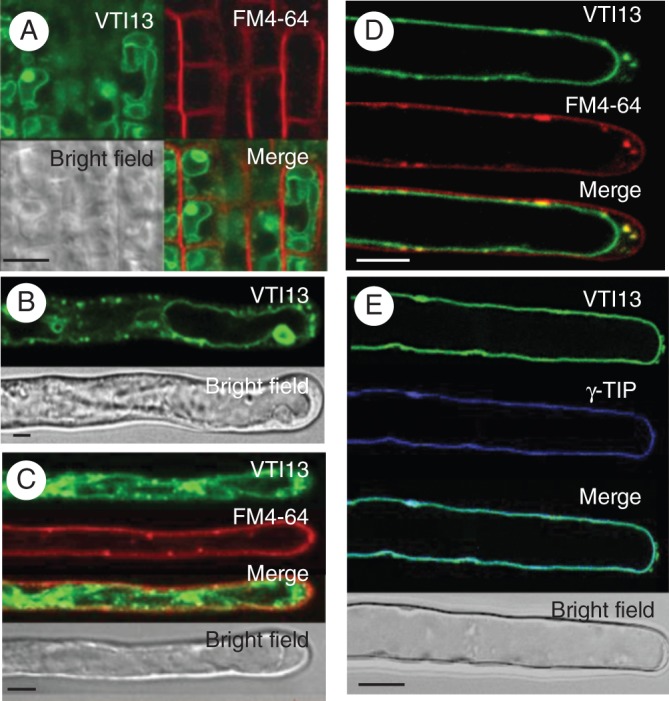
Localization of 35S:GFP–VTI13 in root epidermal cells and root hairs of 5-day-old arabidopsis seedlings. (A) 35S:GFP–VTI13 (green) in root tip epidermal cells highlighting vacuolar ‘bulbs’ and co-stained with FM4-64 (red) for 2–5 min. (B) 35S:GFP–VTI13 in root hairs highlighting vacuolar ‘bulbs’. (C) 35S:GFP–VTI13 (green) in root hairs co-stained with FM4-64 (red) for 2–5 min. (D) 35S:GFP–VTI13 (green) in root hairs co-stained with FM4-64 (red) for 15–20 min. (E) Co-localization of VTI13 (green) and the vacuolar protein YFP-γTIP (purple). The image is a medial section of the z-series through the root hair to show the cross-section view of the cell. Scale bars: (A–D) = 5 μm; (E) = 10 μm.

To identify the intracellular compartments within which VTI13 functions, we characterized GFP–VTI13 subcellular localization in root epidermal cells and root hair cells using confocal scanning laser microscopy (CSLM). While both constructs complemented *vti13*, the 35S:GFP–VTI13-expressing lines yielded higher-contrast images and were used for all subsequent CSLM experiments except when stated otherwise. GFP–VTI13 was observed in two distinct subcellular locations: the membrane of a large internal compartment and smaller compartments that were punctate and mobile (Fig. 3). We also observed GFP–VTI13 fluorescence in vacuolar ‘bulb’-like structures (Fig. 3A, B), which have been characterized previously with respect to the localization of family member VTI11 within vacuolar membranes (Saito, 2003; Saito *et al*., 2011). VTI13 localized to these structures in both epidermal cells at the root tip and in root hairs (Fig. 3A, B).

Due to its intracellular localization in suspension cultured cells (Uemura *et al*., 2004), we wanted to confirm that VTI13 localized to the vacuolar membrane in root hairs and determine if the VTI13 punctate structures represented a class of endosomes. To confirm that the large, VTI13-labelled internal membrane corresponded to the vacuole, transgenic lines expressing 35S:GFP–VTI13 in a wild-type background were crossed with a transgenic line expressing a resident vacuolar membrane protein, YFP-γTIP (Nelson *et al*., 2007). GFP–VTI13 was found to co-localize with the vacuolar marker YFP-γTIP at the vacuole membrane only and not in the small, mobile GFP–VTI13 compartments in growing root hairs (Fig. 3E). These results support a role for VTI13 in the transport or docking of vesicles at the vacuole membrane during root hair growth.

To investigate the nature of the small, punctate VTI13-labelled internal structures, we treated GFP–VTI13 expressing seedlings with the lipophilic dye FM4-64, which is used to label the plasma membrane and internal membranes involved in endosomal trafficking (Fischer-Parton *et al*., 2000). When these seedlings were labelled with FM4-64 for 2–3 min, no co-labelling between FM4-64 and GFP–VTI13 was observed in root hairs (Fig. 3B), indicating that GFP–VTI13 did not localize within the plasma membrane. However, after longer treatments of 15–20 min with FM4-64, we did observe co-localization in some of the GFP–VTI13 mobile fraction within small puncta near the root hair tip (Fig. 3D), suggesting that this might represent a population of endosomal compartments. In these experiments, a similar pattern of localization for GFP–VTI13 was observed in both root tip epidermal cells and root hairs (Fig. 3A, B), indicating that VTI13 localization is similar in these two cell types and that VTI13 is a component of intracellular membranes.

### VTI13 mobility requires the actin cytoskeleton in root hair cells

**Fig. 4.**
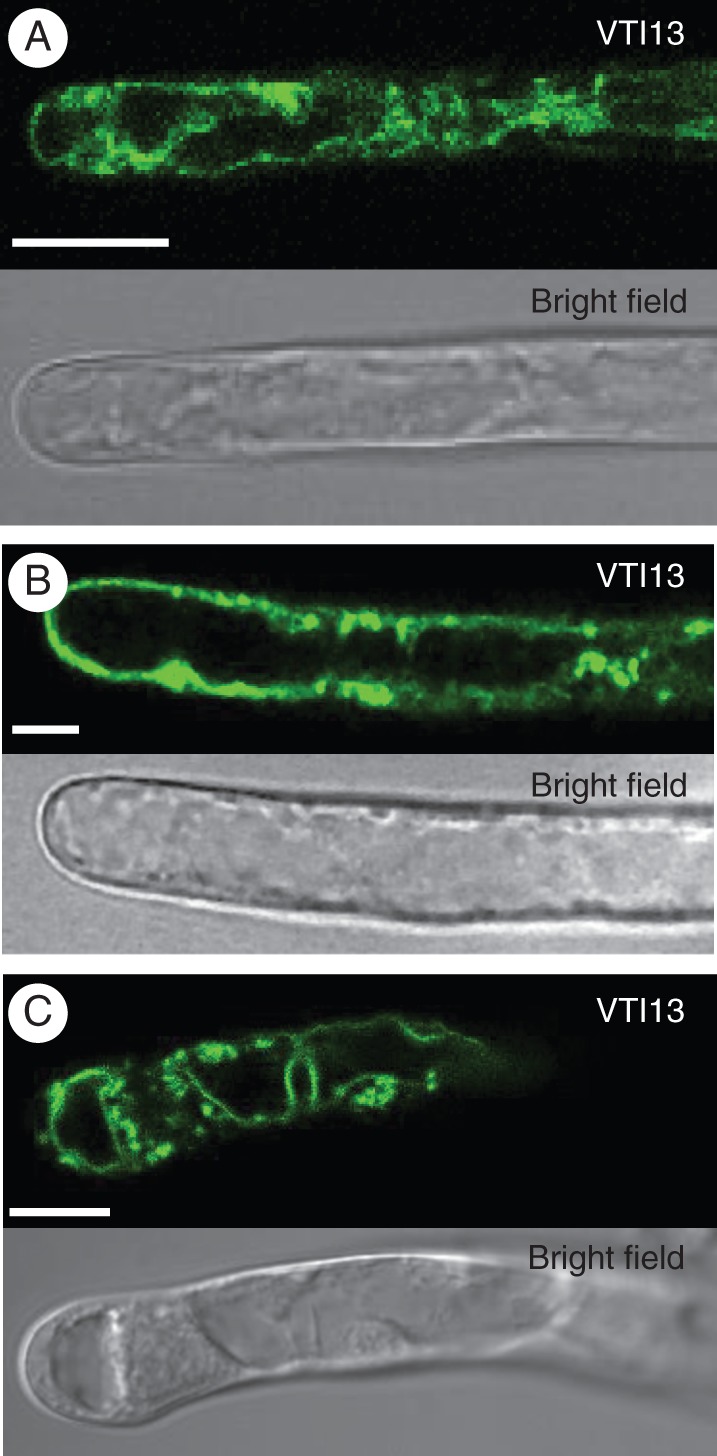
VTI13-labelled compartment mobility requires the actin cytoskeleton. The distribution of VTI13-labelled compartments in the *vti13*-complemented lines was diffuse throughout the root hairs of 5-day-old seedlings treated with (A) 100 nm DMSO or (B) 10 μm oryzalin, and became aggregated after treatment with (C) 100 nm Latrunculin B. All treatments were performed at root temperature for 2 h. Scale bars: (A–C) = 10 μm.

Endocytosis and vesicular trafficking are dependent on the structure of the actin cytoskeleton but not on microtubule organization (Baluška *et al*., 2002; Grebe *et al*., 2003). To investigate whether the mobility of the GFP–VTI13-labelled compartments required an intact actin cytoskeleton, we treated 5-day-old seedlings with 100 nm Latrunculin B, an actin-depolymerizing agent, for 30 min. Root hairs were then examined for the mobility of GFP–VTI13-labelled compartments using CSLM. When seedlings were treated with vehicle alone there was dynamic movement of both the GFP–VTI13-labelled vacuole membrane and the punctate compartments, which moved over long distances along the shaft of the root hair (Fig. 4A, Supplementary Data Video S1). Seedlings treated with 10 μm oryzalin, a molecule that depolymerizes microtubules, had no effect on the movement of the VTI13-labelled vacuolar membrane or mobile compartments in root hairs when compared with DMSO-treated seedlings (Fig. 4B, Supplementary Data Video S2). In contrast, treatment of root hairs with Latrunculin B resulted in the loss of long-distance movement of the small cytoplasmic puncta as well as dynamic changes in the vacuole membrane (Fig. 4C, Supplementary Data Video S3). These data demonstrate that the mobility of the VTI13-labelled vacuole membrane and the small, mobile VTI13-containing compartments requires an intact actin cytoskeleton during root hair growth.

### VTI13 localizes to a population of early endosomes

As a first step in determining the identity of the mobile and punctate GFP–VTI13-labelled compartments in root hairs, we treated seedlings with the fungal toxin BFA. BFA has been shown to selectively inhibit GTP exchange factors (GEFs), which activate ADP ribosylation factors (ARFs) and are required for vesicle trafficking between endosomal compartments in eukaryotes (Peyroche *et al*., 1999; Robinson *et al*., 2008; Lam *et al*., 2009). In plants there are BFA-sensitive and BFA-resistant ARF–GEFs whose localization in different endosomal compartments can be species- and tissue-specific (Richter *et al*., 2007, 2012; Langhans, 2011)*.* While the ARF–GEF GNOM-LIKE1 (GNL1) that is associated with the Golgi apparatus is considered BFA-resistant in arabidopsis roots, concentrations of BFA greater than 50 μm induce a morphological aggregation of TGN containing SYP61–GFP in arabidopsis root protoplasts (Langhans *et al*., 2011). Although the exact mechanisms behind the variety of BFA effects in plants are still being defined, it is well accepted that BFA provides a tool to distinguish between different classes of endosomal compartments.

**Fig. 5.**
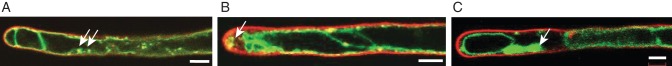
VTI13-labelled compartments are sensitive to BFA. Scanning laser confocal micrographs of 35S:GFP–VTI13 (green) seedlings treated with FM4-64 (red) and (A) DMSO, (B) 10 μm BFA or (C) 50 μm BFA for 10 min only exhibited sensitivity at higher concentrations of BFA. Still frames from each treatment were taken from live images of treated root hairs (Supplementary Data Videos S4–S6). Arrows point to VTI13-labelled compartments whose mobility was not altered by treatment with (A) DMSO or (B) 10 μm BFA, but became swollen and less mobile when treated with (C) 50 μm BFA. Scale bars: (A–C) = 5 μm.

We analysed GFP–VTI13-labelled compartment sensitivity to BFA by treating 5-day-old seedlings with vehicle alone or with a low (10 μm) or intermediate (50 μm) concentration of BFA for 10 min. The seedlings treated with 10 μm BFA for 10 min showed no discernable difference in GFP–VTI13 organization or localization when compared with seedlings treated with vehicle alone, particularly with respect to the mobility of VTI13-labelled compartments (Fig. 5A, B, Supplementary Data Videos S4, S5). In contrast, when seedlings were treated with 50 μm BFA for 10 min, the GFP–VTI13 compartments became larger and less mobile (Fig. 5C, Supplementary Data Video S6). Interestingly, the VTI13-labelled compartments did not co-localize with the endosomal trafficking tracer FM4-64 after BFA treatment. Additionally, when seedlings were treated with 100 μm BFA for 2 h, VTI13-labelled compartments were found to be distinct from those labelled with FM4-64 (Supplementary Data Fig. S3) and were characteristic of the ‘BFA compartments’ thought to be composed of early endosomes and TGN-derived compartments (Geldner *et al*., 2003; Grebe *et al*., 2003). This contrasts with the BFA compartments that did label with FM4-64 in these experiments, which are likely due to the accumulation of endocytosed FM4-64-labelled plasma membrane (Dettmer *et al*., 2006; Richter *et al*., 2012). Together, these data suggest that VTI13 localizes to early endosome or TGN compartments that are sensitive to BFA in a similar manner to that which has been observed in arabidopsis root protoplasts (Langhans *et al*., 2011).

**Fig. 6.**
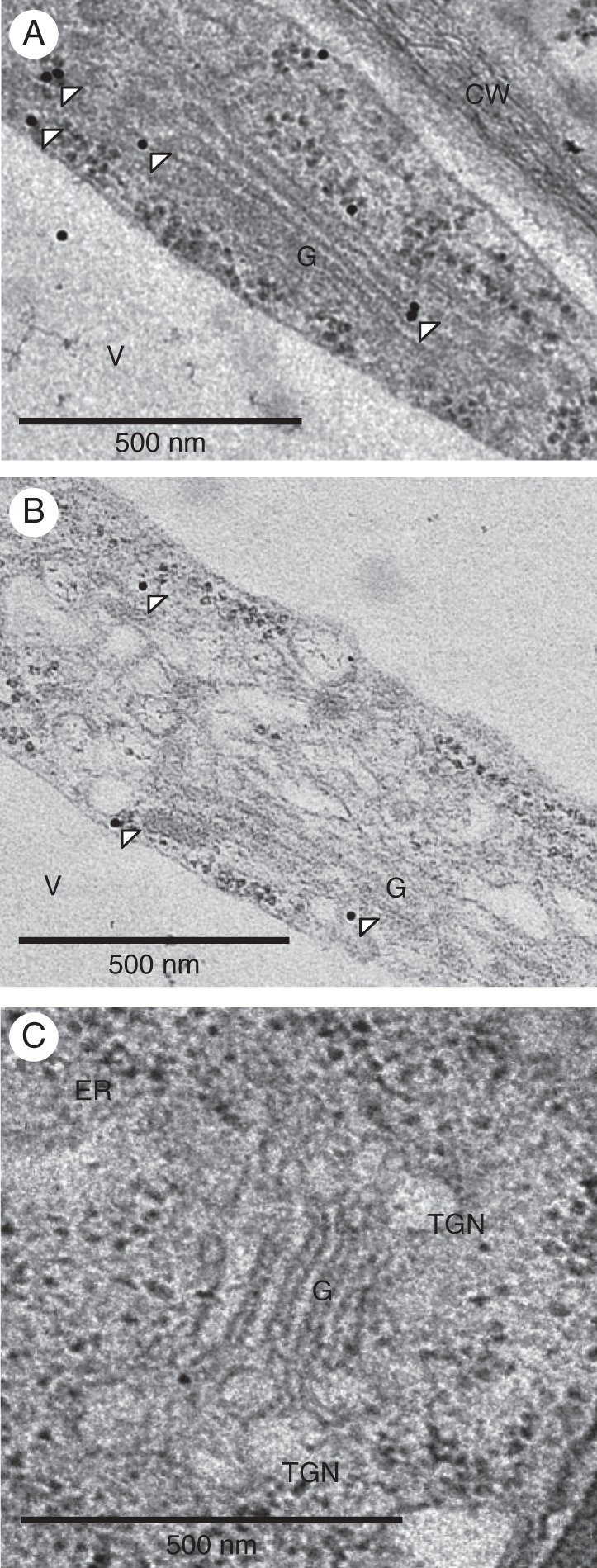
VTI13 localizes to the post-Golgi and *trans*-Golgi network or early endosomes in arabidopsis root hair cells. GFP–VTI13 was visualized using TEM and α-GFP antibodies conjugated to 10 nm gold particles on root hair sections. (A, B) Longitudinal sections of individual root hair cells expressing 35S:GFP–VTI13 show VTI13 localized to the periphery of the Golgi and post-Golgi compartments. White arrowheads point to representative gold particles labelling VTI13 in these subcellular regions. (C) Longitudinal section of a root hair cell expressing 35S:GFP–VTI13 showed no immunogold labelling when only secondary antibody was used. CW, cell wall; V, vacuole; G, Golgi apparatus. Scale bars: (A–C) = 500 nm.

To provide additional support for the localization of VTI13 to early endosomes or TGN compartments, TEM was used with immunogold labelling to visualize the subcellular localization of GFP–VTI13 in root hairs of 5-day-old arabidopsis seedlings. When root hairs of *vti13*-complemented lines expressing 35S:GFP–VTI13 were labelled with an anti-GFP antibody, gold particles were seen at the vacuole membrane, the Golgi apparatus and the periphery of the Golgi stacks (Fig. 6). These data, together with our previous analysis of GFP–VTI13 localization, support a localization of VTI13 within the Golgi and a population of early endosomes or the TGN in arabidopsis root hair cells.

### SYP41 is mislocalized in *vti13* mutant root hairs

**Fig. 7.**
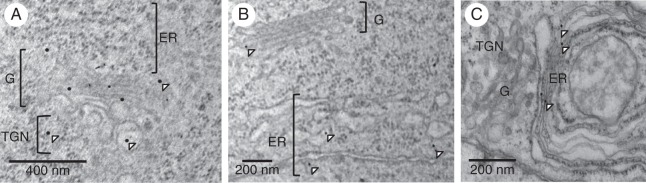
VTI13 function is important for TGN compartment organization. (A) SYP41 antibodies labelled the Golgi and TGN compartments of wild-type root hairs. (B, C) In *vti13* root hairs, SYP41 was predominantly detected in the ER. Arrowheads point to representative gold particles labelling SYP41. G, Golgi apparatus. Scale bars: (A) = 400 nm; (B, C) = 200 nm.

SYP41 is a resident TGN protein and has been used as a marker for TGN compartments in plant cells (Sanderfoot *et al*., 2001*a*, *b*; Uemura *et al*., 2004). To investigate if VTI13 was required for TGN function or organization, we compared SYP41 labelling in roots and root hairs of both *vti13* and wild-type seedlings using immunolabelling and electron microscopy. Interestingly, when we used monoclonal antibodies against SYP41 to label *vti13* root hair sections, we found that the gold particles labelled the endoplasmic reticulum (ER) as well as the TGN, whereas in wild-type root hairs SYP41 labelling was restricted to the Golgi apparatus and TGN (Fig. 7). These data indicate that VTI13 function is important for localization to and/or maintenance of SYP41 within the TGN and suggest that TGN trafficking may be compromised in the *vti13* mutant.

### The *vti13* mutant exhibits an altered cell wall organization

**Fig. 8.**
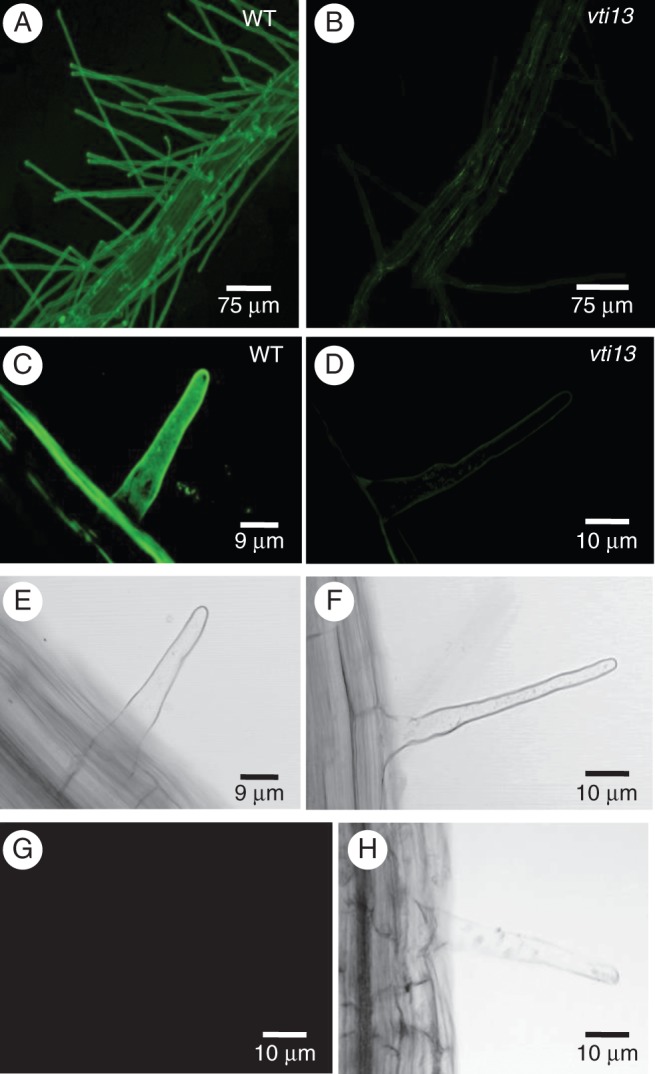
The *vti13* mutant seedling root epidermal cells and root hairs exhibit altered cell wall organization. Confocal microscopy analysis of LM15 labelling in wild-type seedlings shows extensive and uniform distribution of xyloglucan in the cell wall of (A) root epidermal cells and (C) root hairs. Conversely, the *vti13* mutant showed little to no LM15 labelling of the cell wall of (B) root epidermal cells and (D) root hairs. (E, F, H) Bright-field images of root hairs in (C, D and G), respectively. (G) Secondary antibodies alone did not label the cell wall of wild-type root hairs. All images were taken with the confocal microscope at the same settings to show the difference in labelling at the same setting and antibody concentration. Scale bars: (A, B) = 75 μm; (C, E) = 9 μm; (D, F, G, H) = 10 μm.

Our initial observation of altered root hair morphology in the *vti13* mutant suggests that cell wall organization or structure may be affected by *VTI13* expression and function. To test this directly, we used the antibody LM15 (Marcus *et al*., 2008) to label xyloglucan epitopes in root hair cell walls of wild-type and *vti13* mutant seedlings. We hypothesized that if cell wall organization were altered in *vti13* root hairs, we would see a change in LM15 labelling when compared with labelling patterns of wild-type root hairs. Using CSLM, we observed ubiquitous LM15 distribution in the walls of wild-type root epidermal cells (Fig. 8A) and root hairs (Fig. 8C). In the *vti13* mutant, LM15 labelling was minimal along the root epidermis (Fig. 8B) and in root hairs (Fig. 8D). These results suggest that xyloglucan may be underrepresented in *vti13* mutant cell walls or that the organization of *vti13* cell walls has been altered in such a way that the epitope recognized by LM15 is no longer accessible to the antibody. These results support the hypothesis that VTI13 function and/or the pathways in which it functions are important for root hair cell wall organization or structure and that the cell wall of *vti13* root hairs is altered from that of wild-type root hairs.

### VTI13 overexpression may contribute to the prp3 root hair phenotype

**Fig. 9.**
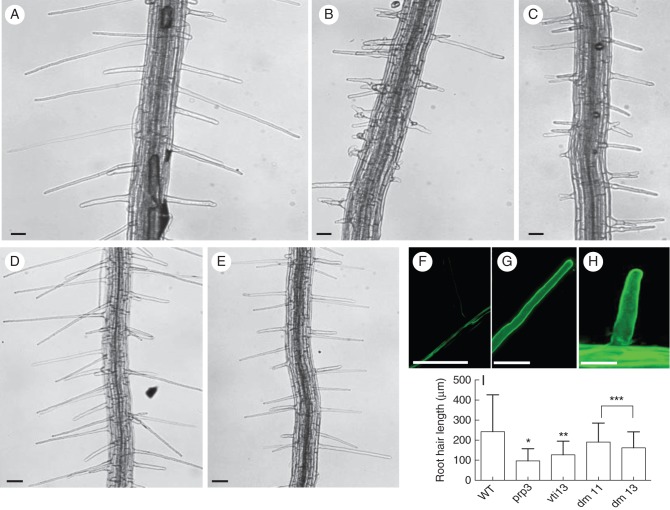
The *prp3 vti13* double mutant suppresses the root hair phenotypes of the single-mutant parents. Comparison of root hairs of 5-day-old (A) wild-type, (B) *prp3*, (C) *vti13* and (D, E) *prp3 vti13* double-mutant seedlings from two independent crosses between *prp3* and *vti13*. (F) The *prp3 vti13* double mutant recovers LM15 labelling in root hairs compared with (G) the *prp3* and (H) *vti13* single mutants, respectively. (I) The *prp3*, *vti13* and double-mutant root hairs are significantly shorter than wild-type root hairs. These data are from a representative specimen with means (±2 s.d.) and asterisks denoting statistical significance (α = 0·01) in average root length when compared with wild-type. Root hairs of approximately eight to ten seedlings were measured for each genotype. Scale bars: (A–E) = 50 μm; (F) = 40 μm; (G) = 50 μm; (H) = 20 μm.

**Fig. 10.**
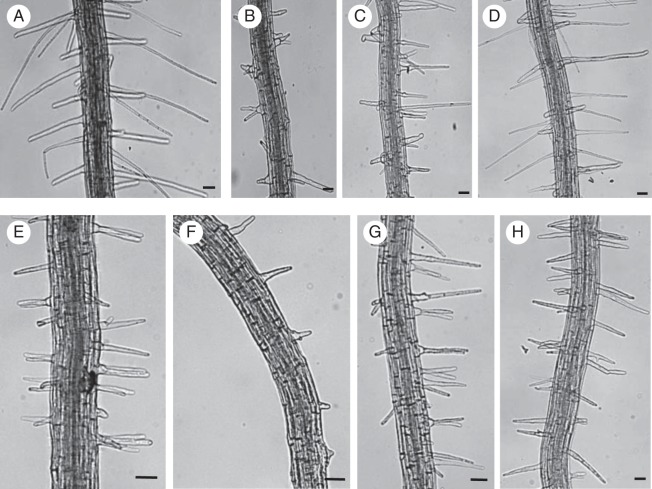
The *prp3 vti13* double mutant was much less sensitive to mannitol in the growth medium when compared with the single mutants and appeared similar in root hair phenotype to wild-type seedlings under both growth conditions. (A, E) Wild type (B, F), *prp3*, (C, G) *vti13* and (D, H) *prp3 vti13* double mutants were grown on 1× MS medium (A–D) and 1× MS medium including 200 mm mannitol (E–H). Scale bars: (A–H) = 50 μm.

The observation that cell wall organization is altered in the *vti13* mutant suggests that VTI13 function may be directly or indirectly involved in cell wall metabolism. Based on our original observation that *VTI13* expression is upregulated in the *prp3* mutant, we were interested in examining if this overexpression might contribute to changes in cell wall structure that would result in the short, branched *prp3* root hair phenotype. If this were the case, then a loss of VTI13 function in the *prp3* mutant background might result in suppression of the *prp3* root hair phenotype. To address this genetically we analysed root hair growth in the *prp3 vti13* double mutant. We found the root hair phenotypes characteristic of the single-mutant parents were partially rescued in the *prp3 vti13* double mutant (Fig. 9). The LM15 labelling of root hairs that was lost in *vti13* was also recovered in the *prp3 vti13* double mutant (Fig. 9F–H). Root hairs of the double mutant no longer branched and were of similar length to wild-type root hairs (Fig. 9I). Additionally, when grown in the presence of 200 mm mannitol, the root hairs of the *prp3 vti13* double mutant responded much more like those of wild-type seedlings than those associated with either of the single *vti13* or *prp3* mutants (Fig. 10). These data demonstrate that VTI13 expression in root hairs is important for normal cell wall organization and suggest that its misregulation in the *prp3* mutant may contribute a change in cell wall organization that is reflected in the observed defects in root hair growth.

## DISCUSSION

In this work we describe the function of VTI13, a SNARE protein that is important for root hair growth. We show that VTI13 localizes to the vacuole membrane and TGN or early endosome compartments in roots and root hairs, is important for TGN integrity and likely plays a role in the transport of cargo to the large vacuole in root hairs. Immunohistochemical analysis indicates that cell wall organization has been altered in *vti13* root hairs, suggesting that VTI13 may play a role in maintaining early endosome trafficking pathways and/or the vacuole function(s) necessary for maintaining cell wall structure. This is supported by suppression of the parental phenotypes and restoration of LM15 labelling of the cell wall in the *prp3 vti13* double mutant.

### VTI13 plays a unique role in root hair growth

Genetic analysis of the arabidopsis *vti13* null mutant identified a short and branched root hair phenotype that could be complemented by expressing two distinct GFP–VTI13 protein fusion constructs individually in the *vti13* mutant background: one driven by the 35S CaMV promoter and one driven by the endogenous *VTI13* promoter. These results demonstrate that the root hair growth defect observed in *vti13* seedlings was due to loss of VTI13 function (Fig. 2). In addition, when these constructs were expressed in a wild-type background, root hair growth was indistinguishable from that of untransformed plants (Fig. 2), indicating that the overexpression of VTI13 alone in growing root hairs, using the 35S promoter, does not compromise endogenous VTI13 function.

The branching root hair phenotype in the *vti13* mutant became more severe when 200 mm mannitol was added to the growth medium (Fig. 1). High concentrations of mannitol result in osmotic stress that can promote plasmolysis and affect growth in root hairs (Lew, 1996; Volgger *et al*., 2010). Mannitol treatment has also been shown to eliminate cellulose synthase complexes from the plasma membrane in non-tip-growing cells, resulting in a change in cell wall assembly during growth (Gutierrez *et al*., 2009). If VTI13 is required for the TGN/early endosome functions necessary for maintaining cell wall metabolism during root hair growth, the elimination of cellulose synthase complexes from the plasma membrane may result in a synergistic phenotype in *vti13* grown in the presence of mannitol. Cellulose synthase subunits associated with primary cell wall assembly are expressed in growing root hairs, although their cellular localization is not associated with the growing root hair tip (Park *et al*., 2011). Thus, the phenotype that *vti13* root hairs exhibit on mannitol-containing media may reflect a synergy between aberrant TGN function and changes in cell wall properties. In contrast to their growth at pH 6, *vti13* mutant root hairs grew long and straight on MS media at pH 5. Oscillations in reactive oxygen species and extracellular pH are correlated with fluctuations in growth at the apex of pollen tubes and root hair cells (Messerli *et al*., 1999; Monshausen *et al*., 2007) and in the extensibility of the cell wall (Monshausen *et al*., 2008). If the altered cell wall organization observed in the *vti13* mutant contributes to its growth phenotype in root hairs, lowering the pH in the growth medium may help relieve aberrant interactions between wall polymers and result in root hairs whose length is more similar to those of wild-type seedlings. While additional experimentation will be required to assess the molecular mechanisms involved in regulating *vti13* root hair growth at different environmental pH, these data suggest that changes in environmental growth conditions may mediate the impact of VTI13 function during polarized growth.

The short, branched root hairs of the *vti13* mutant support a model in which VTI13 is essential for the early endosomal trafficking and/or vacuole function that contribute to polarized growth and cell wall organization in root hairs. This model is supported by the altered xyloglucan organization observed within root epidermal and root hair cell walls in *vti13* when compared with wild-type seedlings (Fig. 8). In addition, analysis of the *prp3 vti13* double mutant allowed us to address whether VTI13 and PRP3 participate in pathways important for cell wall organization in root hairs. The LM15 labelling pattern of the double mutant resembled that of the *prp3* parent and wild-type seedlings (Fig. 9), verifying that loss of LM15 labelling in the *vti13* mutant is due to changes in wall organization. We also observed a more wild-type root hair phenotype in the double mutant than was observed in either of the single-mutant parents. The suppression of the short, branched root hair phenotypes that characterizes both the *prp3* and *vti13* single mutants in the *prp3 vti13* double mutant offers additional evidence that VTI13 functions in a pathway important in maintaining cell wall structure in a manner important for root hair growth in arabidopsis.

### VTI13 localizes to the vacuolar membrane and an early endosomal compartment in arabidopsis root epidermal and hair cells

GFP–VTI13 was found to co-localize with YFP-γTIP at the vacuolar membrane, indicating that VTI13 functions in trafficking pathways to the vacuole. We also observed VTI13 associated with vacuolar ‘bulb’ structures within epidermal cells and growing root hairs (Fig. 3). VTI11 and γTIP localize to vacuolar bulbs and these structures are depleted in the *zig1*/*vti11* mutant (Saito *et al*., 2002). These structures were initially thought to be involved in microautophagy events (Müller *et al*., 2000) but have since been shown to be important for vacuolar function and morphology (Saito *et al*., 2002, 2011; Saito, 2003). Vacuolar function has also been reported to be important for cell expansion (Kroeger *et al*., 2011) and the trafficking of vacuolar cargo may have a meaningful effect on the vacuole's function. The loss of bulb structures in *zig1*/*vti11* is thought to contribute to the disorganized amyloplasts and altered integrity of the vacuole membrane (Saito *et al*., 2011), providing additional evidence that vacuolar structure/function is important for cell growth. If VTI13 localization at the vacuole membrane is required for vacuole function, this could be a contributing factor to the *vti13* root hair growth phenotype. Further investigation is needed to determine if bulb structures or other vacuolar morphologies are compromised and if this contributes to the root hair phenotype we have characterized in the *vti13* mutant.

TEM and immunogold labelling of root hairs in transgenic lines expressing 35S:GFP–VTI13 were used to localize VTI13 to the Golgi and the TGN or an early endosome compartment in root hairs (Fig. 6). The TGN is a known hub for both secretory and endosomal transport and therefore many pathways converge on this compartment (Viotti *et al*., 2010). The localization patterns of the known TGN markers SYP41 and SYP61 only partially overlap, providing evidence for specialization of TGN compartments having different functional identities (Bassham *et al*., 2000; Sanderfoot *et al*., 2001*b*). An additional difficulty in specifically identifying the function of these compartments is the challenge of distinguishing between resident TGN proteins and transient proteins that pass through these compartments as cargo (Hicks and Raikhel, 2010). Recently, dissection of SYP61-containing TGN compartment populations has allowed us to begin a characterization of the proteins that associate with known TGN markers (Drakakaki *et al*., 2011).

Due to the localization of VTI13 to the TGN or an early endosome compartment and the *vti13* mutant root hair phenotype in growing root hairs, we were interested in asking if VTI13 was required for TGN organization. In sections of wild-type root hairs processed for immunogold TEM, we observed SYP41 in the Golgi apparatus and TGN compartments, as expected (Fig. 7A). In contrast, *vti13* mutant root hair sections exhibited SYP41 labelling primarily in the ER (Fig. 7B), suggesting VTI13 function is important for maintaining the identity of SYP41-containing TGN compartments, or that VTI13 may be required to maintain an SYP41 SNARE complex localized within the TGN. Precedence for this comes from work describing TNO1, a SNARE that is important in vacuolar trafficking and that is required for co-localization of SYP61 and VHAa1 (subunit a1 of the vacuolar ATPase) at the TGN (Kim and Basham, 2011). Future experiments will be needed to examine whether VTI13 directly interacts with SYP41 in a SNARE complex that is distinct from that of VTI12 (Bassham *et al*., 2000; Sanderfoot *et al*., 2001*a*) or supports a pathway required for SYP41 localization and function in the TGN.

While localization of VTI13 at the vacuolar membrane in root hairs is consistent with what has been shown for this protein in arabidopsis suspension-cultured cells (Uemura *et al*., 2004), we also observed VTI13 within an early endosome compartment or TGN in root hairs and root epidermal cells (Fig. 3). These differences may be due to several factors, including the use of cell culture versus intact plants as experimental models or the participation of VTI13 in alternative SNARE complexes in cell culture when compared with root epidermal and root hair cells. The localization of VTI13 within the TGN or early endosomes in root hairs is also supported by the co-localization of VTI13 mobile compartments with FM4-64 (Fig. 3D) and the sensitivity of this compartment to BFA. While root hairs treated with 10 μm BFA did not show any difference in GFP–VTI13 localization or VTI13-labelled compartment mobility when compared with DMSO-treated root hairs, we did observe the formation of large, slow-moving VTI13-labelled compartments in root hairs treated with 50 μm BFA (Fig. 5). These results are consistent with VTI13 localizing to the TGN or early endosome compartments and not to the ER or Golgi organelles. In addition, when root hairs were treated with higher doses of BFA for longer periods of time, VTI13 was also found to aggregate into BFA bodies that were distinct from those that accumulated FM4-64 (Supplementary Data Fig. S3). Based on these results, we suggest that VTI13-labelled compartments are unlikely to represent a population of TGN compartments that directly receive cargo from the plasma membrane and traffic to the vacuole via the prevacuolar compartment. Rather, the localization of VTI13 is consistent with compartments that transport cargo between TGN compartments and the internal vacuole in root hairs.

In conclusion, we have defined the localization of VTI13 to the membranes of the vacuole and a population of TGN or early endosomal compartments in arabidopsis root epidermal and root hair cells. We have also characterized the impact of the loss of VTI13 on root hair growth by identifying a branched root hair phenotype in the *vti13* null mutant that is sensitive to environmental growth conditions. Defects in *vti13* cell wall organization are likely to contribute to its root hair growth phenotype, as suggested by altered LM15 cell wall epitope labelling in *vti13* root hairs compared with the labelling pattern in wild-type root hairs. Changes in the cell wall organization may be mediated by alterations in TGN or early endosome function within the *vti13* mutant. We also show that VTI13 is important for the proper localization of SYP41, suggesting a role for VTI13 in maintaining TGN compartment identity through resident protein localization. Further studies will be required to elucidate the mechanism(s) through which VTI13 contributes to cell wall organization and TGN or early endosome function in growing root hairs and the relationship between VTI13 function and the organization of the plant cell wall.

## SUPPLEMENTARY DATA

Supplementary data are available online at www.aob.oxfordjournals.org and consist of the following. Table S1: primers used for genotyping, RT-PCR and cloning of *VTI13* gene sequences. Fig. S1: RT-PCR of *VTI13* and *EF1α* for various amplification cycles. Fig. S2: root hair growth in the *vti13*-complemented line and wild-type in response to mannitol. Fig. S3: reconstructions of z-series stacks of a single root hair showing no co-localization of VTI13 with FM4-64 when treated with BFA. Fig. S4: Settings for imaging dual YFP/GFP-expressing lines. Video S1: transgenic seedling expressing 35S:GFP–VTI13 treated with DMSO for 2 h at room temperature. Video S2: transgenic seedling expressing 35S:GFP–VTI13 treated with 10 μm oryzalin for 2 h at room temperature. Video S3: transgenic seedling expressing 35S:GFP–VTI13 treated with 100 nm Latrunculin B for 2 h at room temperature. Video S4: transgenic seedling expressing 35S:GFP–VTI13 treated with DMSO for 10 min. Video S5: transgenic seedling expressing 35S:GFP–VTI13 treated with 10 μm BFA for 10 min. Video S6: transgenic seedling expressing 35S:GFP–VTI13 treated with 50 μm BFA for 10 min.

Supplementary Data
