# Supplementary material for: SNARE VTI13 plays a unique role in endosomal trafficking pathways associated with the vacuole and is essential for cell wall organization and root hair growth in arabidopsis
Source: Ann Bot. 2014 Apr 15;114(6):1147–59. doi: 10.1093/aob/mcu041 (PMC4195547; doi:10.1093/aob/mcu041)
Supplement: Supplementary Data [file supp_114_6_1147__index.html]

SNARE VTI13 plays a unique role in endosomal trafficking pathways associated with the vacuole and is essential for cell wall organization and root hair growth in arabidopsis — SNARE VTI13 plays a unique role in endosomal trafficking pathways associated with the vacuole and is essential for cell wall organization and root hair growth in arabidopsis — Supplementary Data 

# SNARE VTI13 plays a unique role in endosomal trafficking pathways associated with the vacuole and is essential for cell wall organization and root hair growth in arabidopsis

## Supplementary Data

Supplementary Data

**Files in this Data Supplement:**

- Supplementary Data - Pdf file
- Supplementary Video 1 - mov file
- Supplementary Video 2 - mov file
- Supplementary Video 3 - mov file
- Supplementary Video 4 - mov file
- Supplementary Video 5 - mov file
- Supplementary Video 6 - mov file
