# Supplementary material for: SNARE VTI13 plays a unique role in endosomal trafficking pathways associated with the vacuole and is essential for cell wall organization and root hair growth in arabidopsis
Source: Ann Bot. 2014 Apr 15;114(6):1147–59. doi: 10.1093/aob/mcu041 (PMC4195547; doi:10.1093/aob/mcu041)
Supplement: Supplementary Data [file supp_mcu041_mcu041supp.pdf]

SUPPLEMEANTARY DATA

Table S1. Primers used for genotyping, RT-PCR, and cloning of *VTI13* gene sequences.

**PCR Primers**	**Sequence**
VTI13 Forward	5’-TCTAGATCTATGGAATATGGAAGTTTC -3’
VTI13 Reverse	5’-TCACCTGGTGAGTTTGAAGTACGAGC -3
LB1b	5’-GCGTGGACCGCTTGCTGCAACT-3’
ATPRP3_UPS	5’-GGGCCTATCACATTTTCCGAGCCCAGATAACGTC-3’
ATPRP3_r	5’-GTATCCGTACAACGCCAAGGGAACTCCTG-3’
t-104	5’-CCCCGCAGGGTTGATCACCACTGCCCCCATCTCG-3’
**PCR Product**	**Primer Set**
Endogenous *VTI13* (600bp)	VTI13 Forward, VTI13 Reverse
T-DNA *vti13* (1500bp)	LB1b, VTI13 Reverse
Endogenous *PRP3* (1200bp)	ATPRP3_UPS, ATPRP3_r
T-DNA *prp3* (1500bp)	t-104, ATPRP3_r
	**Cloning Primers**
VTI13pro_F	5’-CCACGATCCGGATCCTGTTTTCTAAATCCA-3’
VTI13pro_R	5’-CCATGGGTTATTTTAATTGTTGCATC-3’

Fig. S1. *VTI13* is expressed at low levels in arabidopsis roots. RT-PCR of *VTI13* and *EF1α* for various amplification cycles. *EF1α* amplification was outside its linear range for all reactions, but *VTI13* amplification in wild type (WT) was only detectable when 40 to 50 cycles were used.

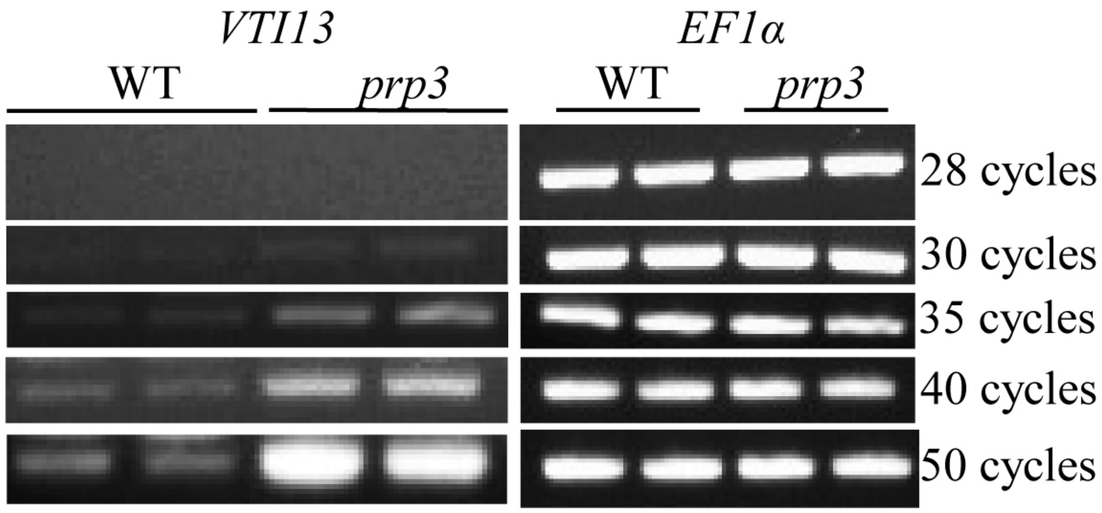

Fig. S2. Root hair growth in the *vti13* complemented line responds to mannitol in a manner similar to wild type. When the T3 seedlings expressing the VTI13:GFP-VTI13 construct in the *vti13* background are grown on MS medium containing mannitol, root hair growth is more similar to wild type than the *vti13* mutant. (A) untransformed wild type grown on 1X MS; (B) untransformed wild type grown on 1X MS plus 200 mM mannitol; (C) untransformed *vti13* grown on 1X MS; (D) untransformed *vti13* grown on 1X MS plus 200 mM mannitol; (E) transgenic seedlings expressing VTI13:GFP-VTI13 line in the *vti13* background grown on 1X MS; (F) transgenic seedlings expressing VTI13:GFP-VTI13 line in the *vti13* background grown on 1X MS plus 200 mM mannitol

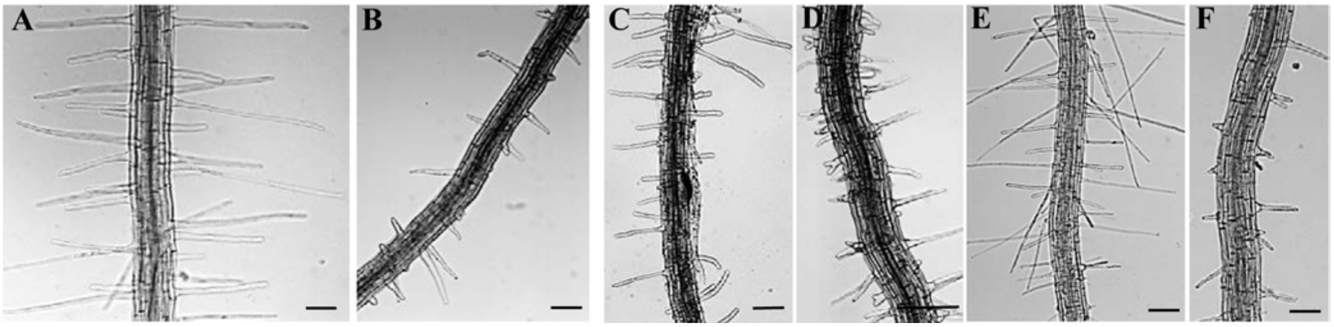

Fig. S3. VTI13 does not co-localize with FM4-64 when treated with BFA. Arabidopsis transgenic seedlings expressing a 35S:GFP-VTI13 construct were treated for 2 hours with 100 μM BFA at room temperature. FM4-64 was added to the BFA solution for the last 20 min of incubation, and seedlings were imaged using confocal microscopy. VTI13 accumulated into large, immobile compartments (filled arrowheads) that did not colocalize with FM4-64-labeled BFA bodies (empty arrowheads). Images are reconstructions of z-series stacks of a single root hair. VTI13 (green), FM4-64 (red). Scale bar = 10 μm.

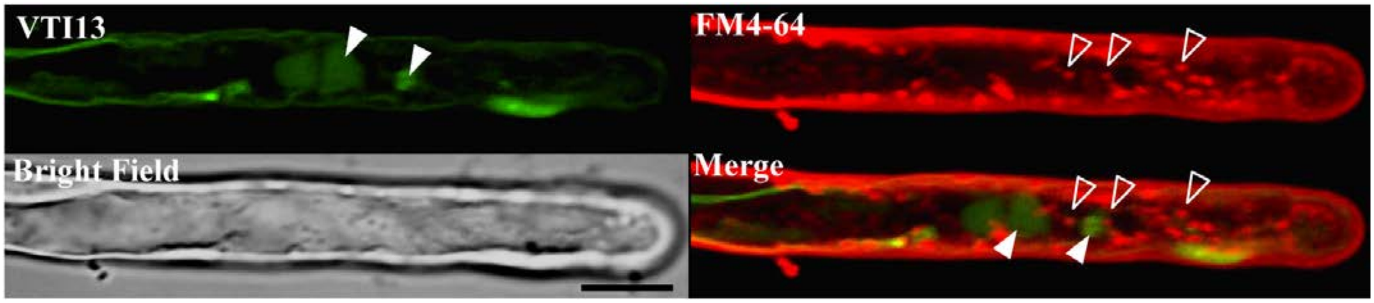

Fig. S4. Settings for imaging dual YFP/GFP expressing lines: The settings used for imaging YFP and GFP constructs together allowed for the separation of both emissions with little to no cross excitation of the two fluorescent proteins. A root hair of a YFP-ER expressing transgenic line imaged to capture (A) GFP or (B) YFP emissions. A root hair of a GFP-VTI13 expressing line imaged to capture (D) GFP or (E) YFP emissions. (C) and (F) are bright field images of ER-YFP and GFP-VTI13 root hairs, respectively. Scale bars = 10 µm.

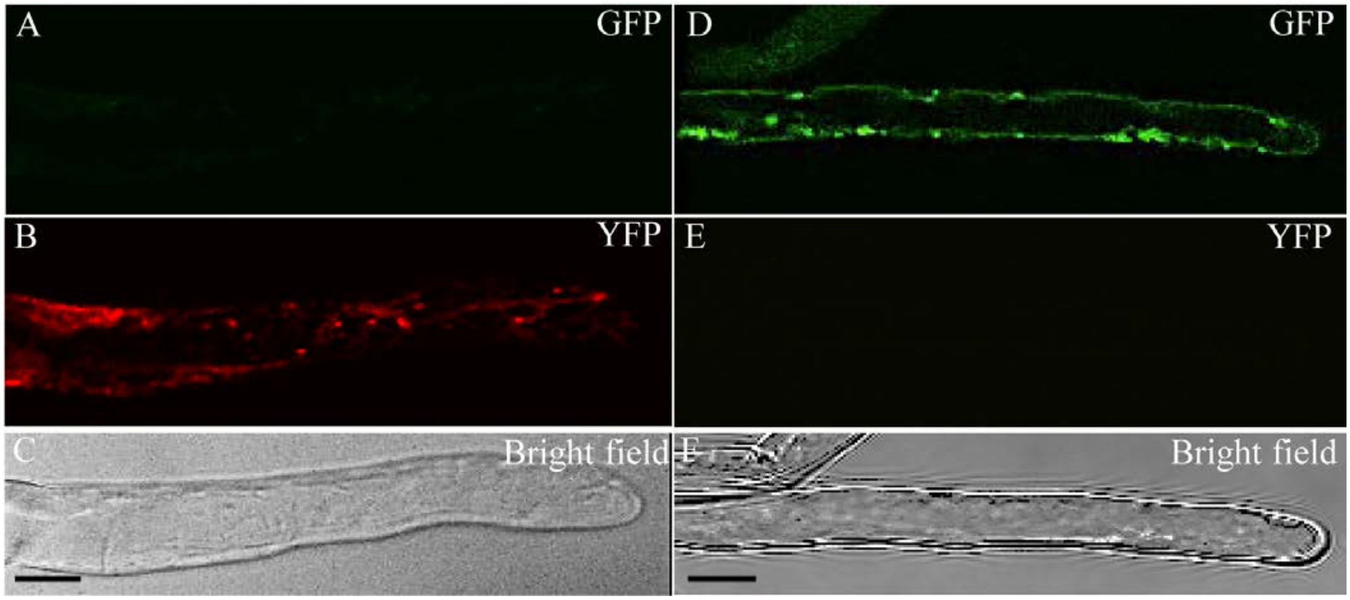

Video S1. Transgenic seedling expressing *35S*:GFP-VTI13 treated with DMSO for 2 h at room temperature.

Video S2. Transgenic seedling expressing *35S*:GFP-VTI13 treated with 10 µM oryzalin for 2 h at room temperature.

Video S3. Transgenic seedling expressing *35S*:GFP-VTI13 treated with 100 nM Latrunculin B for 2 h at room temperature.

Video S4. Transgenic seedling expressing *35S*:GFP-VTI13 treated with DMSO for 10 min.

Video S5. Transgenic seedling expressing *35S*:GFP-VTI13 treated with 10 µM BFA for 10 min.

Video S6. Transgenic seedling expressing *35S*:GFP-VTI13 treated with 50 µM BFA for 10 min.
